# Supplementary material for: Dissociation kinetics and avidity gate SARS-CoV-2 neutralization by HR2 stem helix antibodies
Source: bioRxiv. 2026 Sep 9:2026.08.17.745160. Preprint. [Version 2] doi: 10.64898/2026.08.17.745160 (PMC13622405; doi:10.64898/2026.08.17.745160)
Supplement: Supplementary file 1 [file NIHPP2026.08.17.745160V2-supplement-1.pdf]

bioRxiv preprint doi: https://doi.org/10.64898/2026.08.17.745160; this version posted September 9, 2026. The copyright holder for this preprint (which was not certified by peer review) is the author/funder, who has granted bioRxiv a license to display the preprint in perpetuity. It is made available under a CC-BY 4.0 International license.

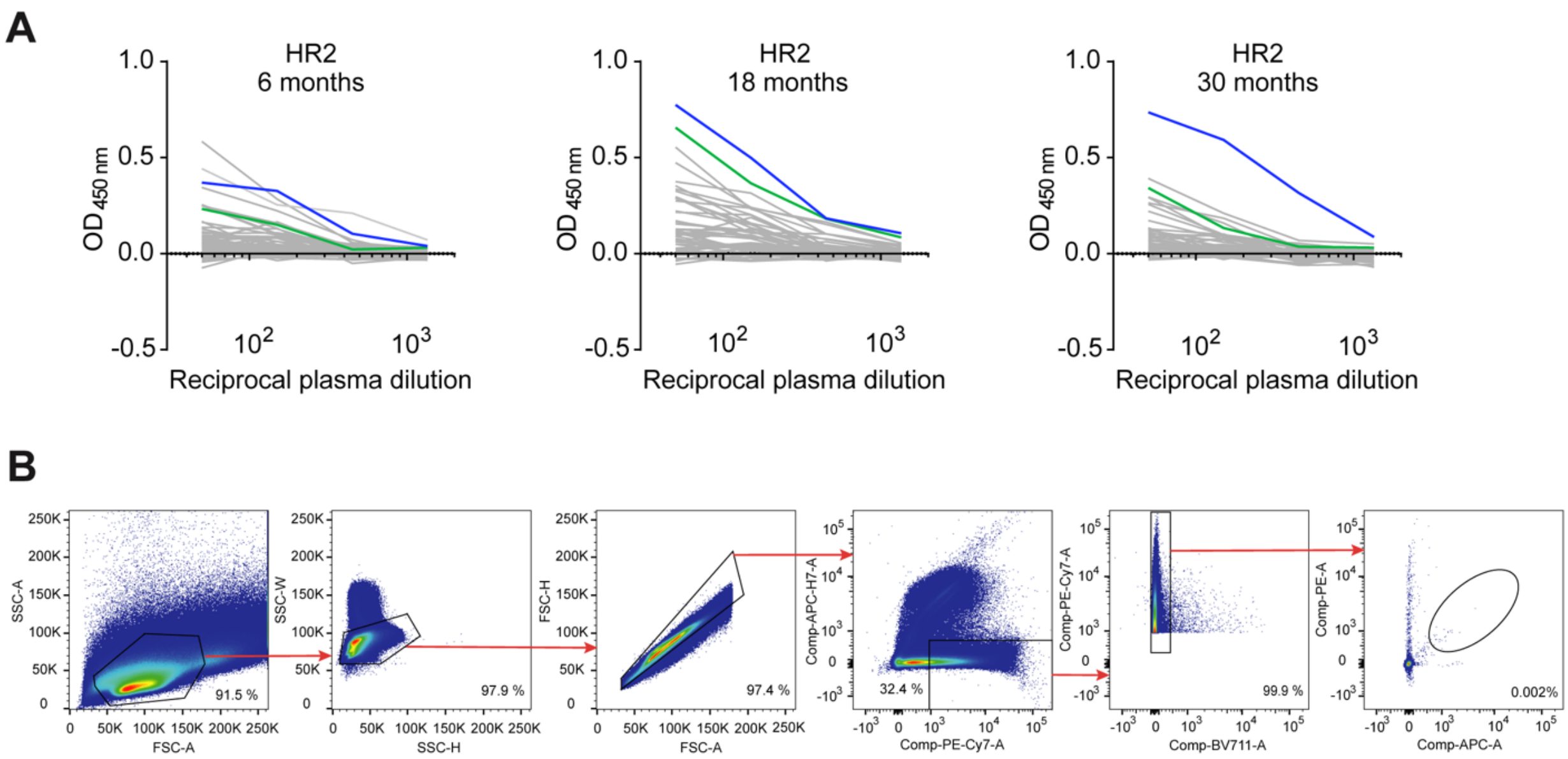

Fig. S1. Longitudinal analysis of HR2 coldspot antibody response. (**A**) ELISAs measuring plasma IgG reactivity to the HR2 coldspot peptide at 6, 18 and 30 months after SARS-CoV-2 infection. Data are shown as mean of two independent experiments. Green (CLM41) and blue (CLM30) represent samples from individuals selected for HR2 coldspot antibody analysis. (**B**) Gating strategy used to sort HR2 coldspot peptide-specific B cells by flow cytometry.

bioRxiv preprint doi: https://doi.org/10.64898/2026.08.17.745160; this version posted September 9, 2026. The copyright holder for this preprint (which was not certified by peer review) is the author/funder, who has granted bioRxiv a license to display the preprint in perpetuity. It is made available under a CC-BY 4.0 International license.

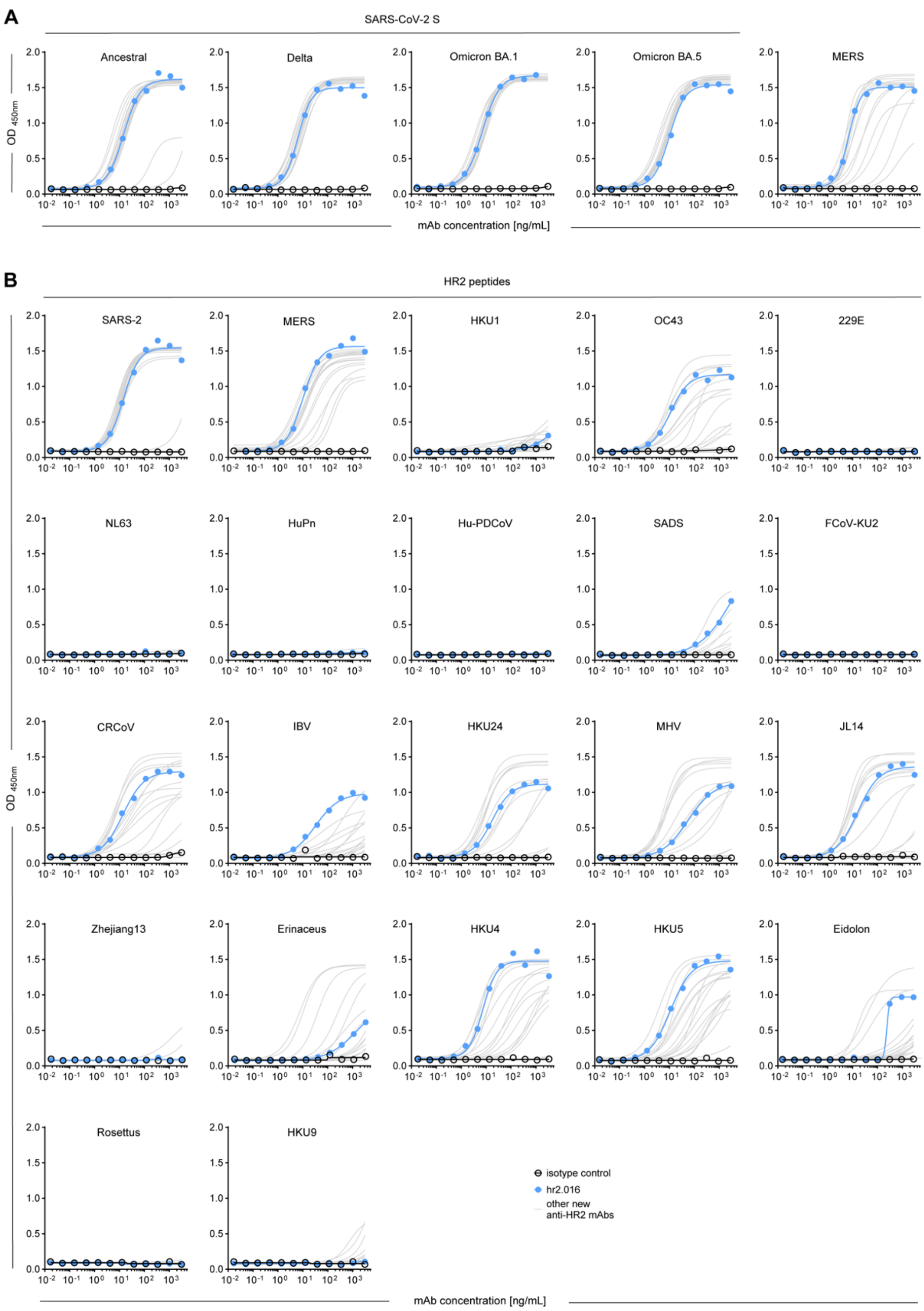

Fig. S2. Crossreactivity of the newly isolated HR2 coldspot antibodies. (**A**) and (**B**) ELISAs measuring HR2 coldspot antibody reactivity to S proteins (A) and HR2 coldspot peptides (B) from different coronaviruses. Data are shown as the mean of two independent experiments.

bioRxiv preprint doi: https://doi.org/10.64898/2026.08.17.745160; this version posted September 9, 2026. The copyright holder for this preprint (which was not certified by peer review) is the author/funder, who has granted bioRxiv a license to display the preprint in perpetuity. It is made available under a CC-BY 4.0 International license.

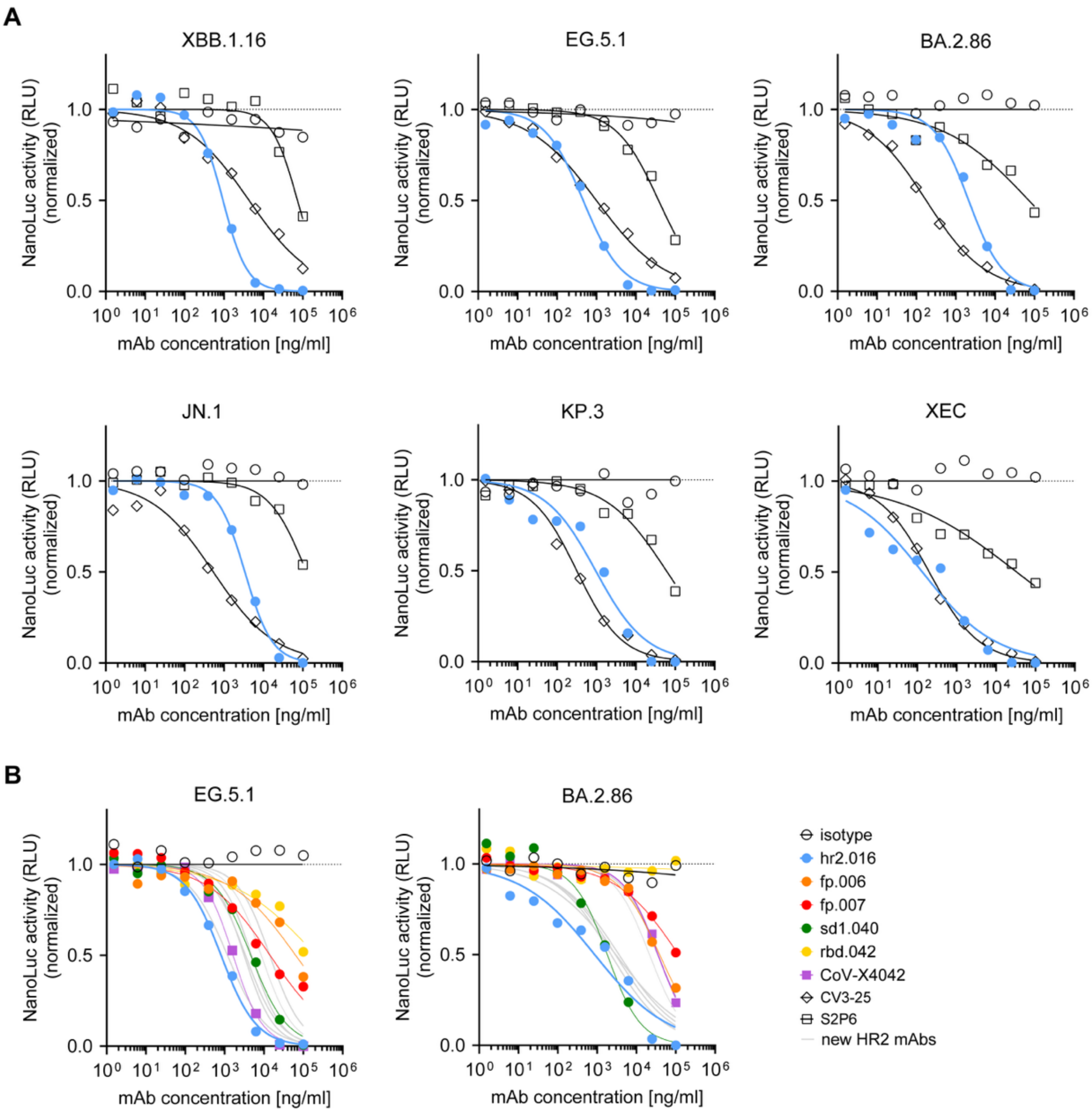

Fig. S3. Broad neutralizing activity of hr2.016. (**A**) Graphs show normalized relative luminescence values measured in cell lysates 48 hours after infection with pseudoviruses of SARS-CoV-2 variants of concern (VOCs) in the presence of increasing concentrations of monoclonal antibodies. An isotype control and the HR2 stem helix antibodies CV3-25 and S2P6 were included for comparison. Data are shown as the mean of two independent experiments. (**B**) Same as (A) but including previously published FP, RBD and SD1 antibodies alongside the newly isolated HR2 coldspot antibodies. Data are shown as the mean of two independent experiments.

bioRxiv preprint doi: https://doi.org/10.64898/2026.08.17.745160; this version posted September 9, 2026. The copyright holder for this preprint (which was not certified by peer review) is the author/funder, who has granted bioRxiv a license to display the preprint in perpetuity. It is made available under a CC-BY 4.0 International license.

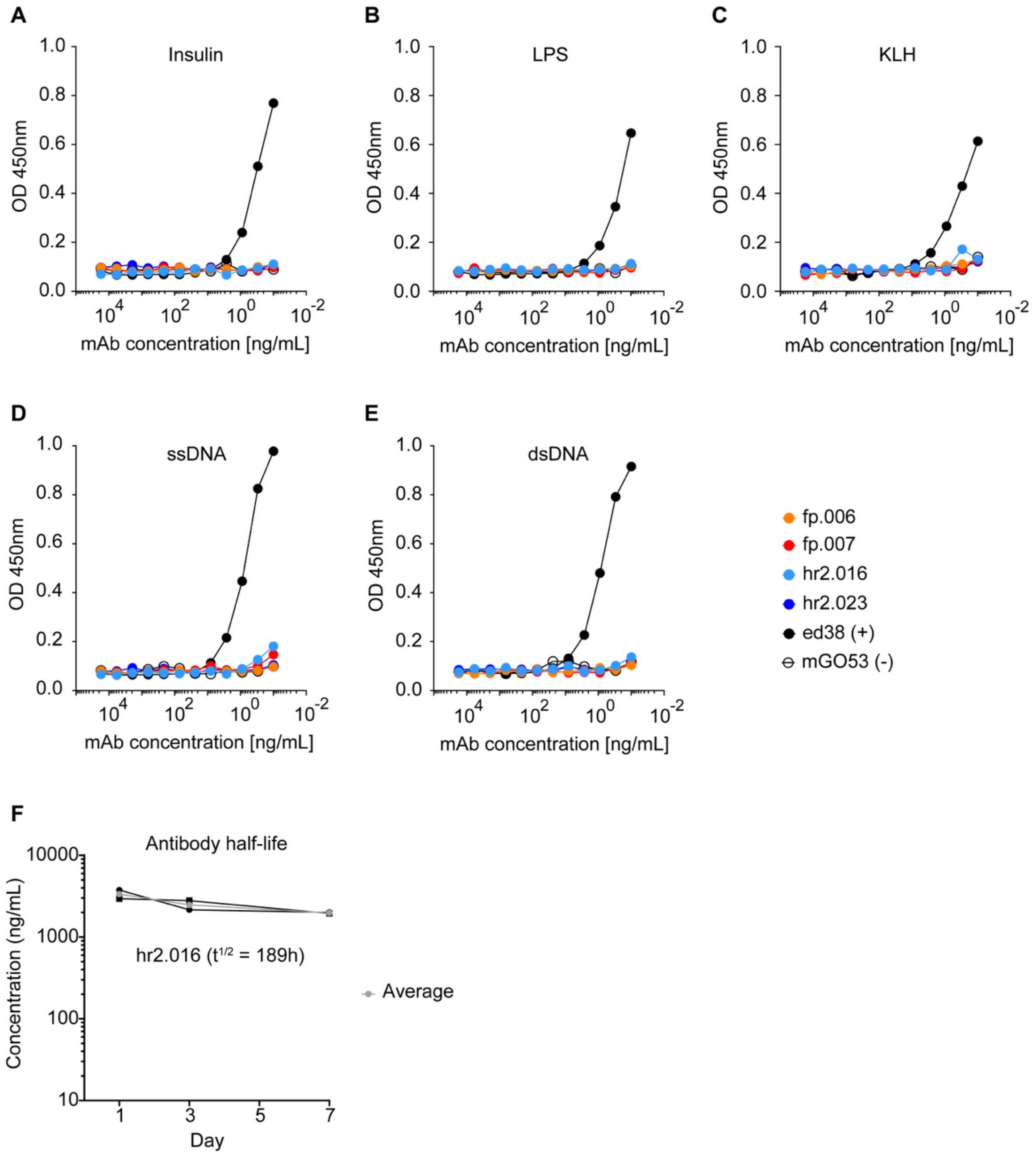

Fig. S4. Developability properties of hr2.016. (**A**) Binding of hr2.016 to insulin, lipopolysaccharide (LPS), keyhole limpet hemocyanin (KLH), single-stranded DNA (ssDNA), and double-stranded DNA (dsDNA), evaluated by ELISA. ED38, a polyreactive monoclonal antibody, was used as a positive control. fp.006, fp.007, and hr2.023 were included for comparison. (**B**) Half-life of hr2.016 in humanized mice following intravenous administration.

bioRxiv preprint doi: https://doi.org/10.64898/2026.08.17.745160; this version posted September 9, 2026. The copyright holder for this preprint (which was not certified by peer review) is the author/funder, who has granted bioRxiv a license to display the preprint in perpetuity. It is made available under a CC-BY 4.0 International license.

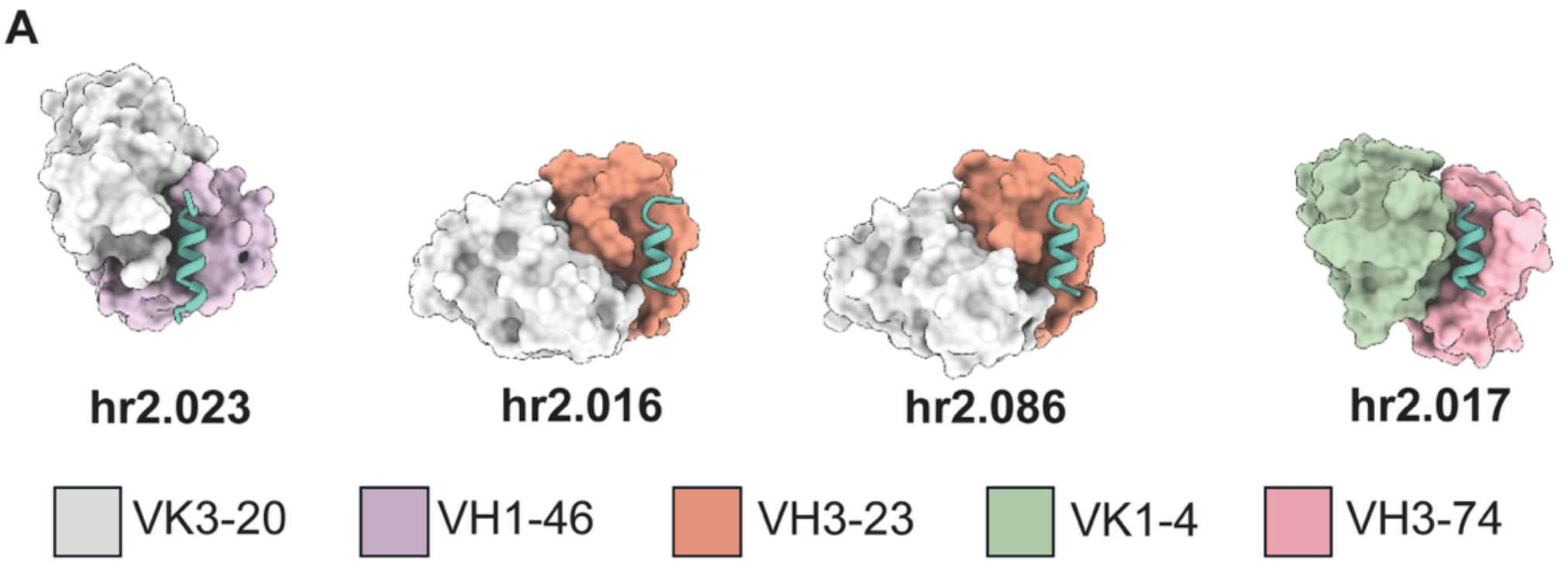

B

		Stem Helix															
Residue Number		1145	1146	1147	1148	1149	1150	1151	1152	1153	1154	1155	1156	1157	1158	1159	1160
Conservation (%)		82	74	83	100	92	82	100	94	84	86	83	96	100	100	81	80
SH Residue		**L**	**D**	**S**	**F**	**K**	**E**	**E**	**L**	**D**	**K**	**Y**	**F**	**K**	**N***	**H**	**T**
BSA (Å²)	**hr2.016**	–	55	77	102	42	0	23	103	30	0	162	131	0	–	–	–
	**hr2.017**	–	–	18	152	121	44	70	57	48	0	112	132	11	–	–	–
	**hr2.023**	38	5	30	148	87	0	65	76	16	2	104	134	11	0	16	25
	**hr2.086**	4	60	61	88	35	0	14	96	34	0	151	129	2	–	–	–
	S2P6	–	4	7	114	47	0	22	61	14	0	74	121	0	0	8	–
	CC40.8	157	92	0	68	72	13	66	109	51	44	102	74	0	5	38	16
	CV3-25	–	0	0	0	14	11	0	23	85	5	0	45	137	55	15	56

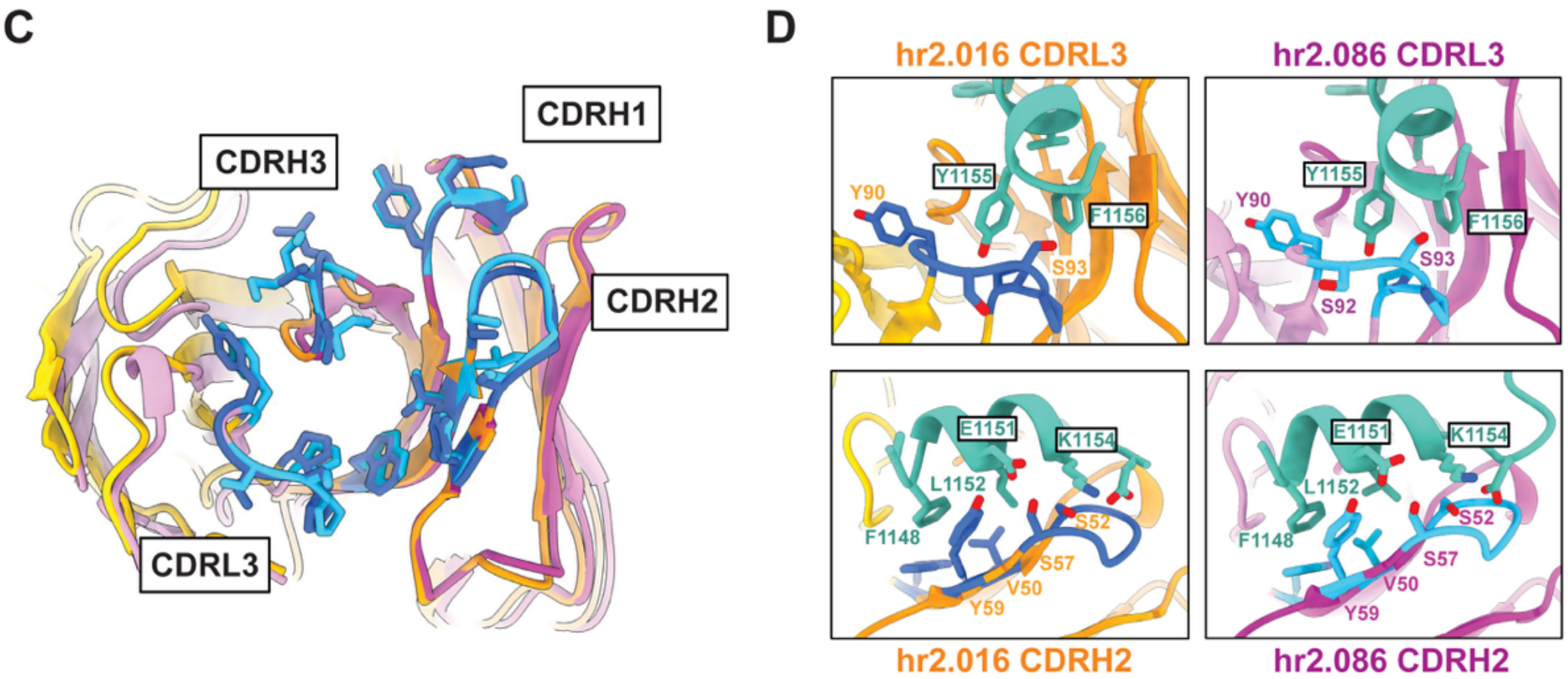

Fig. S5. Structural convergence of antibodies targeting the HR2 stem helix. (A) Crystal structures of four HR2 stem helix-directed antibodies representing distinct germline solutions. Heavy- and light-chain germline gene usage is indicated. **(B)** Buried surface area (BSA) contributed by individual HR2 stem helix residues at the antibody interface for hr2.016, hr2.017, hr2.023, and hr2.086, compared with previously characterized stem helix antibodies S2P6, CC40.8, and CV3-25. Sequence conservation at each HR2 residue across betacoronaviruses is indicated above the heatmap. **(C)** Comparison of hr2.016 and hr2.086 bound to the HR2 stem helix peptide. Paratopes are shaded as blue, with selected antibody residues contributing to the interface shown as sticks. **(D)** Comparison of the CDRL3 (top panels) and CDRH2 (bottom panels) loops of hr2.016 and hr2.086, demonstrating conservation of the principal peptide-contacting residues and interactions between the two clonally related antibodies. Selected interacting residues are shown as sticks.
